# Hypertrophic Cardiomyopathy—Current Challenges and Future Perspectives

**DOI:** 10.3390/jcm12186093

**Published:** 2023-09-21

**Authors:** Emanuele Monda, Giuseppe Limongelli, Francesco Pelliccia

**Affiliations:** 1 Inherited and Rare Cardiovascular Diseases, Department of Translational Medical Sciences, University of Campania “Luigi Vanvitelli”, 80131 Naples, Italy; limongelligiuseppe@libero.it; 2 Department of Cardiovascular Sciences, Sapienza University, 00185 Rome, Italy; f.pelliccia@mclink.it

Hypertrophic cardiomyopathy (HCM) is a myocardial disorder characterized by left ventricular (LV) hypertrophy, which cannot be entirely attributed to loading conditions such as valve or congenital heart disease or hypertension [1,2]. This condition is relatively common, with a prevalence of 1:250–500 individuals, and is linked to increased rates of mortality and morbidity [2]. In recent years, the body of knowledge concerning the genetic underpinnings, natural history, risk assessment, and management of HCM has grown.

In this Special Issue, experts in the field delve into these topics through comprehensive reviews and original articles that explore the molecular basis, the role of genetic testing, risk stratification for sudden cardiac death (SCD), atrial fibrillation, and management of HCM. This Special Issue includes 10 articles, and we are delighted to introduce them to the readers of the Journal of Clinical Medicine.

## 1. Molecular Basis and Genetic Testing

In 40–60% of HCM cases, the disease is inherited as a Mendelian autosomal dominant condition with variable penetrance, associated with pathogenic variants in genes encoding proteins of the cardiac sarcomere [1]. An additional 5–10% of cases are caused by pathogenic variants in genes responsible for conditions mimicking the sarcomeric HCM phenotype [3], such as malformative syndromes (e.g., RASopathies) [4,5,6], storage diseases (e.g., Pompe disease, Fabry disease) [7], infiltrative conditions (e.g., cardiac amyloidosis) [8], and mitochondrial/neuromuscular conditions (e.g., Friedreich ataxia) [8,9,10]. Therefore, genetic testing plays a crucial role in identifying the underlying aetiology and guiding family screening and treatment [9]. 

In this Special Issue, the role of genetic testing in HCM is extensively discussed. Melas et al. describe the main genes implicated in the aetiology of HCM, define the role of diagnostic genetic panels (e.g., next-generation sequencing, whole-exome sequencing, and whole-genome sequencing), and introduce the potential of gene therapy in patients with HCM [10]. In contrast, Lawley et al. emphasize the importance of conducting clinical and genetic screening among paediatric family members of individuals with HCM [11]. Genetic testing should always be prescribed after comprehensive genetic counselling, which aims to educate patients and their families about the benefits and constraints of genetic testing, the genetic aspects related to the disease, and the potential for passing on the condition to their relatives [1]. In their comprehensive review, Girolami et al. address and deliberate on the most commonly posed questions by HCM patients, drawing on their extensive 20-year experience in genetic counselling [12].

## 2. Risk Stratification for Sudden Cardiac Death

SCD has historically been considered the most visible and tragic complication in patients with HCM [13]. Over the past two decades, several risk factors for SCD have been identified, leading to the development of various models and algorithms for the placement of implantable cardioverter-defibrillators (ICDs) in primary prevention [14]. The risk assessment for patients with HCM involves a comprehensive clinical evaluation, which includes clinical and family history, ECG, and imaging tests, with a focus on identifying major risk factors [1,2].

The risk stratification model proposed by the European Society of Cardiology guidelines on the management of cardiomyopathies suggests estimating the 5-year risk of SCD using the HCM-risk score and individualizing ICD implantation based on the estimated risk [1,15]. In contrast, the American College of Cardiology/American Heart Association recommends ICD placement in patients with at least one major risk factor associated with SCD [2,16]. Additionally, specific risk models have been proposed for paediatric patients [17,18].

In this Special Issue, Santoro et al. provide a comprehensive review that summarizes the risk factors associated with SCD in patients with HCM and discusses the different algorithms for ICD implantation [19]. 

## 3. Atrial Fibrillation

Atrial fibrillation (AF) is a prevalent cardiac rhythm disorder in HCM and is linked with increased morbidity and mortality [20]. Risk factors for AF in patients with HCM include advanced age, increased body mass index, disease severity, and left atrial enlargement [20]. 

Sleep apnoea is the most common type of sleep-disordered breathing. Its prevalence among patients with AF is approximately 50% and is likely related to the association between sleep apnoea and left atrial remodelling [21]. However, studies exploring the association between sleep apnoea and AF in HCM individuals are limited. Xu et al. aimed to investigate the association between obstructive sleep apnoea and central sleep apnoea and the prevalence of AF in this condition [22]. The authors observed that 56% of patients had obstructive sleep apnoea and 4% had central sleep apnoea. Additionally, both obstructive and central sleep apnoea were independently associated with AF, indicating the importance of considering sleep apnoea screening as part of AF management in individuals with HCM [22].

## 4. Pharmacological Treatment

The primary objectives of pharmacological treatment in HCM encompass the management of symptoms, lowering dynamic intraventricular gradients, addressing LV systolic dysfunction and HF, managing arrhythmias, and preventing cardioembolic events in patients with AF [23]. 

Beta-blockers are considered the first-line therapy for managing LV outflow tract (LVOT) obstruction [1,23,24]. For individuals experiencing persistent symptoms that are unresponsive or partially responsive to initial treatment, disopyramide may be considered as a secondary therapeutic option [1,23]. Nevertheless, information regarding the safety and effectiveness of disopyramide is limited to a small number of investigations. Maurizi et al. sought to assess the efficacy of disopyramide in addressing LVOT obstruction in HCM patients [25]. Among 118 patients treated with disopyramide, 28 (24%) showed a complete response (defined by reaching the New York Heart Association (NYHA) class I and an LVOT gradient <30 mmHg), 39 (33%) were incomplete responders (defined by a NYHA class >I and an LVOT gradient <30 mmHg), and 51 (43%) were non-responders (defined by no changes in NYHA class and an LVOT gradient >30 mmHg). NYHA class I/II was identified as an independent predictor of response to disopyramide treatment. Eighty patients (69%) showed prolonged QTc interval. However, no major drug-related adverse events were observed. The authors concluded that disopyramide can effectively reduce both LVOT gradients and symptoms in patients exhibiting less severe disease characteristics, all while maintaining a favourable arrhythmia safety profile [25].

Recently, an improved comprehension of the underlying causes of HCM has paved the way for the creation of treatments specifically designed to target the underlying substrate, emphasizing the need for an aetiological characterization of patients with HCM [26]. Thus, Ottaviani et al. furnish a comprehensive overview of the present clinical approaches and delve into emerging therapeutic avenues for sarcomeric HCM, with particular attention to cardiac myosin inhibitors [27].

## 5. Invasive Strategies

Transaortic septal myectomy is considered as the preferred treatment choice for the majority of patients experiencing symptomatic LVOT obstruction that does not respond to medical treatment [2,28]. Myectomy yields instant and enduring elimination of the outflow obstruction and might contribute to atrial and ventricular reverse remodelling [29]. In cases where myectomy cannot be pursued, percutaneous alcohol septal ablation emerges as the most common alternative for addressing LVOT obstruction in HCM patients, with the advantage of shorter hospital stay with a risk of complications similar to myectomy when performed in high-volume centres [2,30]. The decision between myectomy and alcohol septal ablation is based on different variables, including age, comorbidities, centre experience, coronary artery anatomy, and patient preference, among others [2].

Lebowitz et al. provide a detailed review with the aim of discussing the indications of surgical myectomy and alcohol septal ablation, summarizing and comparing the novel techniques for the management of obstructive HCM and offering suggestions for the management of patients with complex presentations [31]. 

In addition, two different articles on the role of alcohol septal ablation are provided in this Special Issue. In their review, Gragnano et al. [32] provide a concise summary of the current evidence concerning alcohol septal ablation. In addition, they underscore the critical importance of assembling a multidisciplinary team of HCM experts comprising clinical and interventional cardiologists, along with cardiac surgeons who have substantial expertise in managing patients with obstructive HCM [32]. Furthermore, Alyaydin et al. investigate the differences related to sex among adults with HCM who undergo alcohol septal ablation. They observed that women tend to present at a more advanced age and with more severe symptoms and that the procedure is safe and effective for both sexes, with advanced age at the time of the intervention as an independent predictor of mortality [33].

The Editors hope that readers of this Special Issue will find it of interest. 
